# Targeting kynurenine metabolism to reduce inflammation and enhance stress response during aging

**DOI:** 10.1093/geroni/igab046.2565

**Published:** 2021-12-17

**Authors:** George Sutphin, Hope Dang, Luis Espejo, Raul Castro-Portuguez, Bradford Hull, Jeremy Meyers, Emily Turner, Destiny DeNicola

**Affiliations:** 1 The University of Arizona, Tucson, Arizona, United States; 2 University of Arizona, Tucson, Arizona, United States; 3 UNIVERSITY OF ARIZONA, TUCSON, Arizona, United States; 4 University of Arizona, Arizona, United States

## Abstract

Aberrant kynurenine pathway metabolism is increasingly linked to aging and age-associated disease. Kynurenine metabolic activity increases with age and becomes dysregulated during various forms of age-associated pathology in humans. By manipulating one or more kynurenine pathway enzymes and metabolites, we have extended lifespan up to 40% in Caenorhabditis elegans. In particular, elevating physiological levels of the kynurenine pathway metabolite 3-hydroxyanthranilic acid (3HAA) by directly supplementing 3HAA or inhibiting the enzyme 3HAA dioxygenase (HAAO) extends C. elegans lifespan by ~30%. 3HAA delivered chronically in chow similarly extends lifespan in aged C57BL/6 mice. In ongoing work, we are investigating the mechanisms underlying the benefits of multiple kynurenine pathway interventions using tools in C. elegans, mice, and human cell culture. We have preliminary evidence for activation of broad-spectrum cellular stress response, enhanced immune function, and reduced inflammation. Among other roles, the kynurenine pathway is the sole metabolic route for de novo synthesis of nicotinamide adenine dinucleotide (NAD+) from tryptophan in Eukaryotic cells. We are examining the regulatory interaction between kynurenine metabolism and the two NAD+ recycling pathways, Salvage and Preiss-Handler, both as potential mechanistic mediators and as possible parallel targets for combined interventions with synergistic benefits in aging. We are further evaluating the impact of these interventions in several models of specific age-associated diseases, including sepsis, chronic inflammation, stroke, Alzheimer’s disease, and cancer. Finally, we are developing pharmaceutical strategies to replicate key genetic and metabolic interventions within the kynurenine pathway that can be readily translated into clinical applications.

